# ABO and Amelogenin Determination by PCR, from Experimental Bloodstains and from Museum Specimens, Using a Non-Destructive Approach

**DOI:** 10.3390/genes17060659

**Published:** 2026-06-04

**Authors:** Tadeusz Dobosz, Małgorzata Bonar, Anna Jonkisz, Natalia Kantyka, Agnieszka Dobosz

**Affiliations:** 1 Department of Forensic Medicine, Division of Molecular Techniques, Wroclaw Medical University, M. Sklodowskiej-Curie 52, 50-369 Wroclaw, Polandanna.jonkisz@umw.edu.pl (A.J.);; 2 Department of Human Biology, Faculty of Biological Sciences, University of Wroclaw, S. Przybyszewskiego 63, 51-148 Wrocław, Poland; malgorzata.bonar@uwr.edu.pl; 3 Department of Basic Medical Sciences and Immunology, Division of Basic Medical Sciences, Faculty of Pharmacy, Wroclaw Medical University, Borowska 211, 50-556 Wroclaw, Poland

**Keywords:** museum specimens, ABO typing, PCR, multiplex, LOH, MSI

## Abstract

Background/Objectives: Museum collections constitute valuable material for investigating a wide range of histological processes. This results from the historical selection of unusual and advanced disease cases by museum curators, which are rarely encountered in contemporary clinical practice due to advances in medicine. Ancient DNA plays a crucial role in phylogenetic studies, as well as in analyses of population genetics. However, many commonly used DNA extraction techniques involve partial degradation of samples prior to DNA isolation. The use of non-destructive methods may enable the recovery of DNA appropriate for downstream analyses. Non-destructive methods of DNA extraction for research purposes are a recent development and facilitate genetic analyses of museum collections. ABO and Amel are examples of applications of the proposed method, although any set of primers can be used. ABO genotyping has been widely used in phylogenetic and population analyses. Methods: This study presents a non-destructive approach for PCR-based DNA extraction from preserved museum samples. Human tissue samples, filter materials used during preservation, and processed conservation fluids (after dilution and dialysis) were analyzed to determine ABO genotype and sex (based on Amelogenin). Results: The same replicable PCR profile (ABO blood group and sex determined by Amelogenin) was observed across all three sample types: tissues, filter papers, and conservation fluid. The use of a preservative solution is a new development, as it leaves the sample intact. However, this approach has a drawback: DNA diffuses into the preservative solution very slowly, and it takes several decades to reach a sufficient concentration. A major advantage of this approach is the ability to perform a PCR test without DNA preparation. Conclusions: Museum-derived samples represent a reliable source of DNA and can be effectively used in PCR-based analyses. The presented method works well with degraded DNA samples, combining an already established very short Amelogenin amplicon with PCR sequence-specific primers for ABO genotyping.

## 1. Introduction

Museum-derived specimens represent an important source of material for studying the various histological phenomena. Ancient DNA makes it possible to trace evolutionary transformations over time and constitutes an important tool in phylogenetic and population genetic investigations [1,2,3,4]. In addition, such material contributes valuable information about the history of human populations. At the same time, working with museum specimens is challenging, as they usually contain highly fragmented and degraded DNA. Therefore, specialized tools are required when processing this type of material. An additional challenge associated with museum specimens is fact that they are valuable for exploring human history and the surrounding world. A major limitation of widely used DNA extraction techniques is that they often rely on prior tissue degradation, which is not suitable when dealing with preserved museum materials. The use of non-destructive methods enables the recovery of DNA suitable for downstream analyses, including PCR, SNP analysis, and sequencing [3,5,6,7,8,9].

It is important to consider the input DNA and its quality when designing subsequent experiments. ABO genotyping has been widely used in phylogenetic and population analysis studies, as well as in forensic testing and transplantation medicine, since the 1990s. Genotyping methods for the ABO groups have been published numerous times, but only the method described by Yamamoto et al. has proven reliable. The molecular basis of the ABO system was elucidated by Yamamoto et al. who identified, at the molecular level, the nucleotide sequences of the six ABO genotypes A1, A2, A1B, A2B, B, and 0, demonstrating the precise glycosyltransferase set that assigns the A, B, and 0 epitopes [10,11].

The ABO blood group system was first described by Karl Landsteiner in the early 1900s [12,13]. Subsequently, Alfred von Decastello and Adriano Sturli described the fourth blood group, AB [14].

Later, in 1939, Philip Levine and Rufus Stetson reported the existence of the Rhesus (Rh) blood group system, including the Rh factor. They distinguished Rh-positive individuals (Rh^+^), characterized by the presence of the D antigen, from Rh-negative individuals (Rh^−^), who lack this antigen, and described the clinical implications associated with unrecognized Rh incompatibility [15].

It was first found by L. Hirszfeld and E. von Dungern that blood group types are inherited in a classical Mendelian manner; however, Felix Bernstein established the correct blood group pattern in 1924 [16,17].

After specific modifications, the ABO system subsequently became widely applied in phylogenetic and population analysis, forensic analysis (e.g., bloodstain analysis and identification of human remains), paternity testing, and blood transfusion or transplantation medicine. Following the introduction of the “mixed agglutination technique”, the type of tissue used and the age of the tested individuals became unimportant, except in the neonatal period, since blood group antigens are not detectable in newborns but emerge during early childhood.

ABO genotyping is advancing with improvements in PCR methods and the discovery of more specific DNA-allele sequencing methods. Eight major common blood group phenotypes are recognized: A1, A2, A1B, A2B, B, 0, Rh^+^, and Rh^−^; however, there are more subgroups than A1 or A2 that differ with a single nucleotide substitution, making hundreds of individual and family genetic variants [18]. PCR methods have been used for the above application since 1995 by Sirnsek et al. [19] to rapidly perform Rh D genotyping. Seltsam et al. [20] examined the complete genomic sequence for diversity and diversification at the ABO locus in 2003, and Jiang et al. [21] developed an integrated multiplex PCR system to simultaneously test for ABO and the core CODIS STRs.

In the present study, attempts were made to isolate PCR-amplifiable DNA from museum specimens older than 100 years using a non-destructive method. Furthermore, the PCR test dedicated to the museum samples, given the difficulties encountered when working with ancient DNA, was designed. Although other blood group systems provide greater discriminatory power than the ABO system, the widespread familiarity with the groups in this system partly compensates for its lower polymorphism. While PCR-based ABO genotyping and Amelogenin analysis are well-established in forensic genetics, the novelty of the present study lies not in the markers themselves but in the non-destructive source of DNA and the multiplex design tailored for 100+ year-old museum specimens preserved in ethanol-glycerol mixtures. Previous non-destructive approaches have relied on surface swabbing or tissue soaking [7,22]. Here, we demonstrate for the first time that original preservation fluid (after decades of storage) and filter paper used during fluid filtration can serve as reliable, amplifiable DNA sources without any damage to the original specimen. Additionally, we show identical ABO and Amelogenin profiles obtained from three different materials derived from the same specimen: tissue, filter paper, and dialyzed conservation fluid. Such cross-validation across three sources has not been reported for historical museum collections. Our approach is not intended to compete with forensic STR or SNP kits for individual identification, but rather to enable population genetic studies and non-destructive screening of valuable museum samples prior to more costly analyses such as massively parallel sequencing [2,5]. Standardized guidelines for working with ancient and museum-derived DNA have been established since the early 2000s, emphasizing dedicated laboratory facilities, physical separation of pre- and post-PCR areas, stringent contamination controls, and independent reproducibility of results [2,6,22,23]. This study follows these principles, as detailed in the Section 2.

A multiplex PCR assay with high analytical sensitivity was developed. First, the constructed multiplex PCR enabled population genetic analyses using museum samples. It could replace the serological method or be used in addition to assays that test for the core CODIS STR loci (or more) to increase discriminatory power and “recycle” the old, now largely useless worldwide data (for instance, accessible from the fundamental monograph by Mourant [24]).

## 2. Materials and Methods

### 2.1. Samples

#### 2.1.1. Museum Specimens

**Figure 1:**
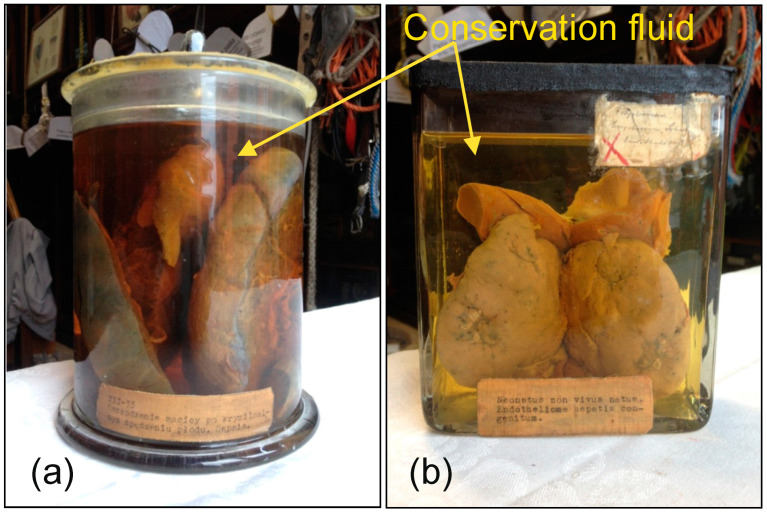
Representative museum specimens used in this study. (**a**) Jar labeled “III-33–Criminal damage to the uterus after expulsion of the fetus, with sepsis”; specimen age approximately 81 years. (**b**) Jar labeled “Newborn, not born alive because of a hereditary tumor of the liver”; specimen age > 100 years. Both specimens were preserved in a water-ethanol-glycerol mixture (30% ethanol, 10% glycerol) without formaldehyde. Scale bars indicate approximate container dimensions (height ~15 cm).

The analyzed material originated from the collections of the Museum of Forensic Medicine, located in the Department of Forensic Medicine at Wroclaw Medical University, and comprised various human tissues. The samples were dated to be over 150 years old. In addition to tissue samples, filter papers used during the filtration of preservation solutions, as well as diluted and dialyzed conservation fluids, were also subjected to analysis. DNA from human tissues and filter papers was extracted using a commercial assay based on the column-spin method. All specimens were stored in their original containers under room temperature conditions with limited light exposure. The filter paper (Whatman GF/C), applied for the filtration of aged, turbid, and dark conservation fluids, was kept at −20 °C until further processing. The preservation medium consisted of a mixture of water, ethanol (30%), and glycerol (10%). The examples of the specimens are presented in Figure 1. These specimens were selected because they were suitable for methodological optimization and allowed limited sampling without compromising collection integrity.

#### 2.1.2. Blood Stain Preparation

Twelve bloodstain samples were obtained from Polish Caucasian volunteers, in affiliation with our local Transfusiology Unit, where ABO blood groups were previously determined using classical serology methods prior to genotyping. Blood samples were collected from the cubital vein, deposited onto filter paper, allowed to dry at room temperature, and subsequently stored in paper envelopes for one year under standard laboratory conditions.

### 2.2. Primer Design

The sequence of ABO primers [21] along with fluorescence dyes are listed in Table 1. Forward primers were tagged at the 5′ end with two fluorophores (FAM and JOE). The amplified fragments covered a size range of 50–100 bp without overlapping. The same rules were applied to construct two home-made STR kits, internal named “Pentaplex” and “Hexaplex”.

The primers for ABO typing were designed according to Jiang et al. [21] and target four diagnostic nucleotide positions that distinguish alleles A, B, and 0. Forward primers are labeled at the 5′ end with either FAM (for 0 and A alleles) or JOE (for AB, B and Amelogenin). The expected amplicon sizes are as follows: 0 allele–78 bp and 92 bp; A allele–82 bp and 92 bp; B allele–82 bp and 97 bp. The amplicon length for Amelogenin is 208 bp (X chromosome) and 214 bp (Y chromosome), enabling sex determination. The short length of ABO amplicons (50–100 bp) was deliberately chosen to maximize success rates with highly fragmented ancient DNA. Amelogenin primer sequences are included in Table 1.

### 2.3. DNA Extraction

#### 2.3.1. DNA Extraction Using 5% Chelex^®^ Suspension

Bloodstain fragments of approximately 2 mm were excised and transferred into 1.5 mL Eppendorf tubes containing 1 mL of distilled water. The samples were incubated at room temperature for 15–30 min, followed by centrifugation at 13,500 rpm for 3 min. The supernatant was subsequently removed and discarded. Next, 180 µL of 5% Chelex^®^ suspension was added to each tube containing approximately 4 mm^2^ of the bloodstain material. The samples were incubated at 56 °C for 20 min, and then heated at 98 °C for 8 min. After incubation, the tubes were centrifuged again at 13,500 rpm for 5 min. Samples were stored at 4 °C and only used for a two-week period, after which a new extraction was carried out.

#### 2.3.2. DNA Extraction Using NucleoSpin^®^ Tissue Column

Samples consisted of either 25 mg of tissue or approximately 1 cm^2^ of filter paper used during filtration of the preservation fluid. The material was fragmented into smaller pieces and transferred into microcentrifuge tubes. Subsequently, 180 µL of Buffer T1 and 25 µL of Proteinase K were added to ensure complete coverage of the sample. The mixture was vortexed and incubated at 56 °C for 90 min in a shaking incubator set to 600 rpm to ensure complete tissue lysis. All subsequent steps were performed according to the manufacturer’s protocol for the NucleoSpin^®^ Tissue kit (Macherey-Nagel, Düren, Germany). DNA was eluted in 50 μL of Buffer BE supplied with the kit. No additional purification, concentration, or quantification step was applied to these extracts. For amplification, 4 μL of the column eluate were used directly in the PCR reaction (see Section 2.3.4). DNA extracts were stored at −20 °C.

#### 2.3.3. Dissection of DNA Presented in the Conservation Fluid Sample

50 µL of filtered conservation fluid was pipetted into the 8 mm diameter Visking Dialysis Tube (membrane) and dialysed at 4 °C, on a magnetic stirrer. The process was carried out for 12 h in PCR buffer (100 mM Tris, 500 mM KCl, 0.1% (*v*/*v*) Tween 20, and 2 mM MgCl_2_, adjusted to pH 8.35 with HCl), followed by an additional 12 h dialysis step in deionised water. After dialysis, 4 µL of the prepared sample was added to the PCR reaction mixture prior to amplification. DNA concentration was determined using a Qubit Fluorometer (Invitrogen/Thermo Fisher Scientific (Waltham, MA, USA)).

Dialysis was chosen to remove low-molecular-weight PCR inhibitors (e.g., ethanol, glycerol, and phenolic compounds) that co-diffuse from the preserved tissue into the conservation fluid over decades. The choice of 12 h in PCR buffer followed by 12 h in deionized water was based on preliminary optimization experiments, which showed that shorter dialysis (e.g., 6 h) resulted in incomplete inhibitor removal (manifested as failed PCR amplification), while longer dialysis (>24 h) did not improve DNA recovery but increased the risk of DNA loss through the membrane (pore size ~12–14 kDa cutoff). The PCR buffer composition (100 mM Tris, 500 mM KCl, 0.1% Tween 20, 2 mM MgCl_2_, pH 8.35) was selected because it matches the final PCR conditions, allowing direct addition of 4 µL of dialyzed fluid to the amplification reaction without further adjustment.

The dialyzed conservation fluid (4 µL) was added directly to the PCR reaction mixture (Section 2.3.4) without any column purification step, as the dialysis membrane (12–14 kDa cutoff) effectively removed inhibitors while retaining DNA fragments >~50 bp.

##### Contamination Control and Reproducibility

All DNA extraction and PCR setup steps were performed in a dedicated ancient DNA laboratory physically separated from post-PCR facilities, under UV-sterilized laminar flow hoods. Extraction blanks (mock extractions without sample) and PCR negative controls (nuclease-free water instead of DNA template) were included in every amplification run. No amplification product was observed in any negative control. Following established ancient DNA guidelines [2,6,23], the following additional contamination control measures were implemented: (i) all surfaces were cleaned with 10% bleach and 75% ethanol, followed by UV irradiation for at least 30 min before each experiment; (ii) disposable filter tips and sterile consumables were used exclusively; (iii) researchers wore full protective clothing including masks, gloves, and dedicated lab coats; (iv) post-PCR products were never introduced into the pre-PCR facility; (v) multiple negative controls (extraction blanks and PCR blanks) were included in each run to monitor contamination at every step. To assess reproducibility, each museum specimen was analyzed in duplicate from independent extractions, and whenever possible, from two of the three available source types (tissue, filter paper, or preservation fluid). A profile was considered interpretable only when the same ABO and Amelogenin alleles were detected in at least two independent PCR reactions from the same specimen.

##### Interpretation Criteria

The following criteria (Table 2) were adopted for accepting ABO and Amelogenin genotypes in museum specimens.

These criteria were applied consistently across all sample types (tissue, filter paper, and conservation fluid).

#### 2.3.4. Amplification

A primer mix was prepared by diluting 1 μL of each ABO and Amelogenin forward and reverse primer (100 μM each) with 40 μL of TE buffer to obtain a final concentration of 2 μM. For each PCR reaction, 0.8 μL of this primer mix was added to 5 μL of Qiagen^®^ Multiplex PCR Master Mix, 0.2 μL of nuclease-free water, and 4 μL of DNA template–either from the column eluate (Section 2.3.2, for museum tissue/filter paper) or from the dialysate (Section 2.3.3, for conservation fluid). The total reaction volume was 10 μL. PCR amplification was carried out using a BioRad™ S1000 thermal cycler (Bio-Rad Laboratories (Hercules, CA, USA)) under the following conditions: initial denaturation at 95 °C for 15 min; 30 cycles of denaturation at 94 °C for 30 s, annealing at 63 °C for 60 s, and extension at 72 °C for 45 s; followed by a final extension at 72 °C for 10 min. Samples were then held at 4 °C.

#### 2.3.5. Electrophoresis, Detection, and Analysis

Capillary electrophoresis and analysis were carried out using the ROX500 Internal Size Standard (50–500 bp) in accordance with the manufacturer’s protocol (Applied Biosystems). PCR products (1 µL) were mixed with 10 µL HiDi formamide containing the size standard and denatured at 95 °C for 5 min, then immediately cooled at 0 °C for 3 min. Separation was performed on an ABI Prism 3130 Genetic Analyzer (Applied Biosystems/Thermo Fisher Scientific, Foster city, CA, USA) equipped with POP-4 polymer and a 36 cm capillary. Injection was conducted at 10 kV for 3 s, and fragments were resolved at 15 kV at 60 °C.

PCR products were analyzed directly without dilution unless peak signals exceeded the detector’s linear range.

## 3. Results

### 3.1. Museum Specimens DNA Extraction

DNA was extracted from museum-derived materials (including both tissues and filter papers) using a spin-column-based approach. In contrast, the filtered preservation fluids were subjected to dialysis at 4 °C prior to further analysis. A total of twenty museum specimens were included in this study. The DNA concentration obtained from human tissues was 20 ± 2.5 ng/μL. From conservation fluids, DNA concentrations of 10 ± 3.1 ng/µL were obtained, whilst from filtered paper, the obtained DNA concentration was about 5–10 ng/μL.

#### 3.1.1. Sensitivity and Reproducibility

To determine the analytical sensitivity of the assay, DNA extracted from a control bloodstain (known group A, male) was serially diluted from 10 ng/µL to 0.1 ng/µL. Full ABO and Amelogenin profiles (i.e., all expected peaks with RFU > 150) were obtained down to 0.5 ng/µL. At 0.2 ng/µL, allele dropout was observed in 2 out of 6 replicates (33%). Therefore, the practical sensitivity limit of the assay was set at 0.5 ng/µL input DNA. In all 12 bloodstain samples (modern controls), duplicate PCRs yielded 100% concordant ABO genotypes matching the serological reference. Among the 20 museum specimens, triplicate analyses from the same specimen (across tissue, filter, and/or fluid) showed 100% concordance for both ABO and Amelogenin. Extraction blanks and PCR negatives remained negative throughout.

#### 3.1.2. Amplification Success Rate

The amplification success rate was defined as the percentage of PCR reactions yielding a full interpretable profile (all expected ABO and Amelogenin peaks with RFU ≥ 100, according to Table 2). Across all 20 museum specimens and three source types (tissue, filter paper, conservation fluid), a total of 180 PCR reactions were performed (20 specimens × 3 sources × 3 replicates). The overall success rate was 88.3% (159/180). Success rates by source type were: tissue–95.0% (57/60); conservation fluid–86.7% (52/60); and filter paper–83.3% (50/60). The lower success rate for filter paper and conservation fluid likely reflects lower DNA yields from these sources (5–10 ng/µL) compared to tissue (20 ± 2.5 ng/µL). Allele dropout (defined as the absence of one expected allele in a heterozygous sample) occurred in 3.9% (7/180) of reactions; all dropouts were observed at DNA concentrations below 0.3 ng/µL, consistent with the sensitivity limit established in Section 3.1.1.

### 3.2. The Blood Stain Analysis

A total of 12 blood-stain samples were used in the analysis to adjust the genotyping method. In all samples, the ABO alleles and sex-determination loci were consistently detected. Notably, the ABO genotype corresponded to the ABO blood group determined by the local Transfusiology Unit using the classical serological method. The size distribution of PCR products was distinct, with no overlap, allowing unambiguous differentiation. Genotyping of the ABO system resulted in four possible amplicon sizes (78, 82, 92, and 97 bp), depending on the specific genotype, as summarized in Table 2.

For blood group B, two or four fragments were detected: homozygous BB samples produced two products (82 and 97 bp), whereas heterozygous B0 samples yielded four fragments (78, 82, 92, and 97 bp).

In the case of blood group A, homozygous AA individuals generated two fragments (82 and 92 bp), whereas heterozygous A0 samples produced three products (78, 82, and 92 bp).

Group 0 was identified by the presence of two fragments (78 and 92 bp), whereas the AB genotype was characterized by three fragments (82, 92, and 97 bp).

The amplicons generated for the Amelogenin marker were longer than those obtained for ABO. Male samples produced two distinct signals (209 and 215 bp), whereas female samples showed a single fragment of 209 bp.

The fragment sizes corresponding to both ABO and Amelogenin markers are summarized in Table 3.

**Figure 2:**
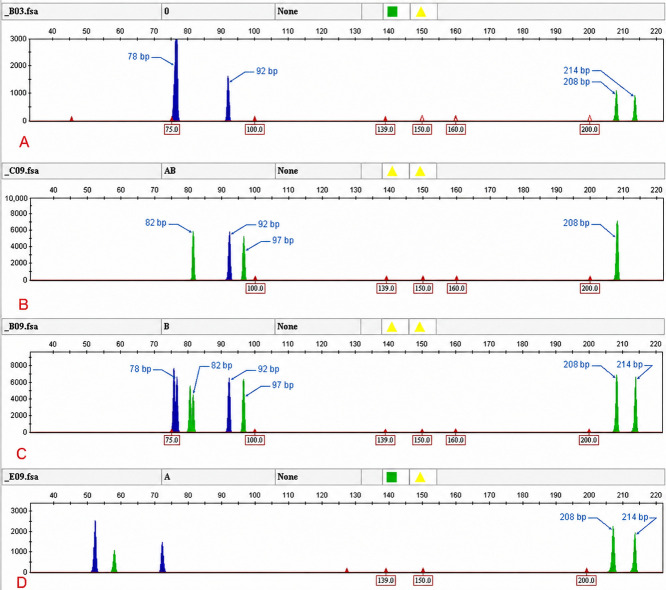
Representative electropherograms of ABO blood groups and sex determination (Amelogenin) obtained from bloodstain samples (modern controls, *n* = 12). Panels: (**A**) blood group 0 (male, 78 bp and 92 bp); (**B**) blood group AB (female, 82 bp, 92 bp, and 97 bp); (**C**) blood group B0 (male, 78 bp, 82 bp, 92 bp, and 97 bp); (**D**) blood group A0 (male, 78 bp, 82 bp, and 92 bp). Amelogenin: male samples (**A**,**C**,**D**) show two peaks at 208 bp (X) and 214 bp (Y); female sample (**B**) shows a single peak at 208 bp (X). Arrows indicate allele peaks. All profiles were fully concordant with serological typing.

The electropherograms showing ABO alleles 0, AB, B0, and A0, and Amelogenin sex determination, with both male and female blood stain samples, are presented in Figure 2.

### 3.3. Museum Specimens Analysis

**Figure 3:**
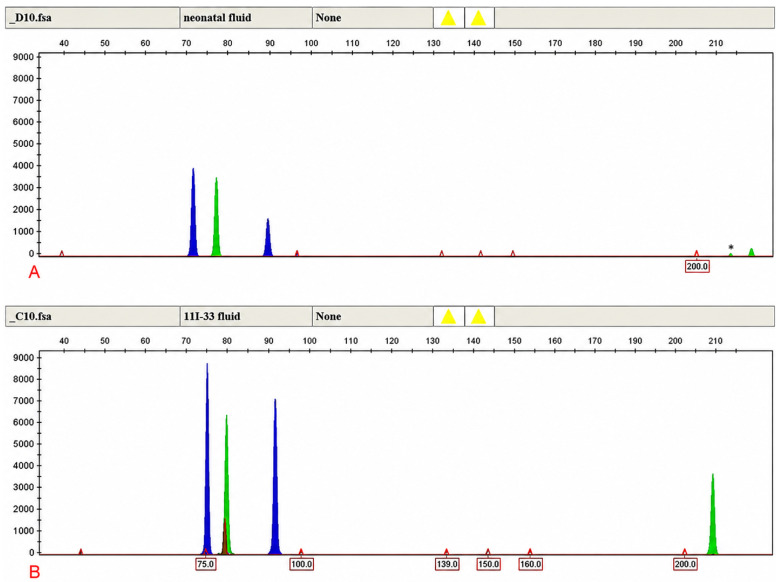
Electropherograms showing ABO* blood group and sex (Amelogenin) in museum specimens. Panel (**A**) male newborn (specimen from Figure 1b); note the microsatellite instability (MSI) indicated by an asterisk (*) and loss of heterozygosity (LOH) type pattern for Amelogenin. Panel (**B**) female specimen III-33 (specimen from Figure 1a) with a regular ABO and Amelogenin profile. In cancer-derived tissues such as these historical specimens, MSI and LOH may be observed as incidental findings. Fragment sizes are indicated above peaks. The presence of MSI in the newborn specimen (Panel (**A**)) is consistent with the known diagnosis of a hereditary tumor (hepatoblastoma).

After adjusting the PCR conditions based on known blood groups, the assay was used to determine the ABO genotype and sex in museum specimens. The assay yielded reproducible amplification profiles on the biological material and on the filter paper from the museum samples when processed using NucleoSpin^®^ Tissue Column. The assay was also successfully applied to dialysed, filtered conservation fluid, producing reproducible and interpretable amplification profiles. Complete genotyping profiles were obtained. across all analyzed museum specimens for the ABO, along with Amelogenin (as shown in Figure 3).

**Figure 4:**
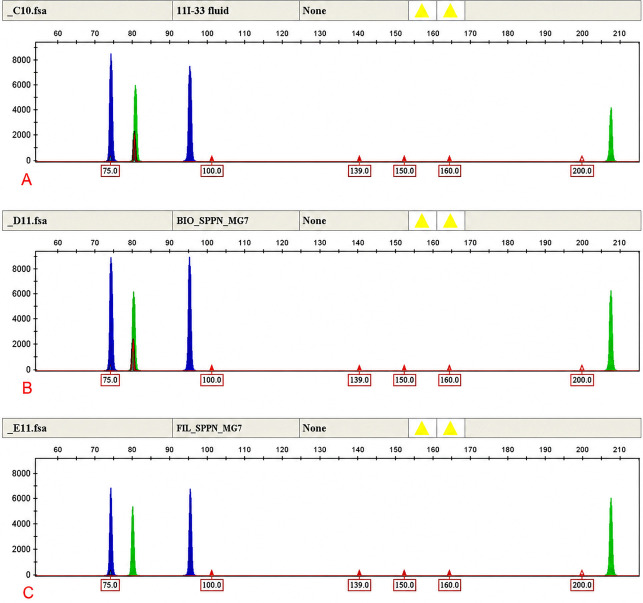
Electropherogram profiles of ABO group A (A0 genotype) and female sex (Amelogenin) obtained from three different sources derived from the same museum specimen (III-33, >150 years old). (**A**) Dialyzed conservation fluid; (**B**) tissue; (**C**) filter paper used during fluid filtration. Tissue and filter paper were processed using the NucleoSpin^®^ Tissue Column protocol; the conservation fluid was processed by dialysis. The short amplicon lengths (ABO: 78–97 bp; Amelogenin: 208 bp) enabled successful amplification despite advanced sample age. Note the identical profile (A0, female) across all three sources, demonstrating the reliability of non-destructive DNA recovery from associated materials.

The specificity of multiplex PCR assay was additionally evaluated by analyzing three different materials derived from the same museum specimen: tissue, filter paper, and preservation fluid. Comparable and reproducible PCR patterns (ABO genotyping and sex identification using Amelogenin) were observed in all sample types, as illustrated in Figure 4 [25].

The relatively short PCR amplicons enabled successful profile generation in all analyzed samples.

## 4. Discussion

This study analyzed DNA isolated from museum tissues and associated materials, including filter paper and conservation fluid. Despite partial degradation, PCR amplification was successful. Furthermore, a multiplex PCR system for ABO blood group typing and sex determination was presented, which is rapid and cost-effective. Despite partial DNA degradation, the ABO assay successfully amplified all targeted loci.

Museum specimens can be an excellent tool for studying evolutionary changes. However, there are significant technological limitations in handling this material using molecular methods [1]. The challenges of working with museum specimens, particularly regarding contamination control and authentication of results, have been formalized in published guidelines since the early 2000s [2,6,23]. Key requirements include physical separation of pre- and post-PCR areas, use of multiple negative controls, replication of results from independent extractions, and, when possible, independent replication in a different laboratory [2,8]. The presented study meets these requirements through the measures described in Section Contamination Control and Reproducibility and through independent replication across three different source materials (tissue, filter paper, and preservation fluid) from the same specimen. A major limitation associated with museum-derived DNA is the degradation, chemical modification, and/or contamination of DNA. Various strategies for isolating DNA from museum specimens have been described in the literature, aiming to enhance both the quantity and quality of the extracted genetic material; however, their effectiveness has been inconsistent [26]. The effects varied depending on numerous conditions, such as the age of the museum specimens and the preservation agent. Successful DNA recovery has previously been reported for samples ranging from 20 to over 70 years old [27,28]. In the present paper, DNA from 100-year-old museum samples was successfully obtained and performed well in the PCR assay. A major technical challenge was the presence of ethanol- and glycerol-derived PCR inhibitors, which interfered with the PCR assay. All three DNA purification approaches applied in this study yielded successfully purified DNA. The column-based extraction method enabled the recovery of DNA free from ethanol and glycerol. Furthermore, using a 5% Chelex-based method that relies on sample heating at 98 °C purified the DNA by evaporation of ethanol and other contaminants. The filtration approach used to process the preservation fluid further removed ethanol and glycerol via dialysis. The effectiveness of these procedures was confirmed by successful PCR amplification.

While our study focused on successful PCR amplification rather than detailed aDNA damage analysis, it is important to acknowledge that DNA from century-old museum specimens is expected to exhibit characteristic damage patterns, including cytosine deamination (resulting in C→T transitions) and depurination leading to strand breaks [29,30]. Such damage could potentially affect primer binding and amplification efficiency. However, the short amplicon lengths (50–100 bp) used in our multiplex were deliberately chosen to minimize the impact of fragmentation, as previous studies have shown that aDNA fragments are predominantly <150 bp [2,5]. The successful amplification across all three source types suggests that although damage was likely present, it did not completely preclude PCR at these short target sizes. Future work could apply massively parallel sequencing to directly assess damage patterns in DNA recovered from preservation fluid, which would provide additional authentication criteria following established aDNA guidelines [22,31].

Museum specimens are generally rare and scientifically valuable materials. Conventional DNA extraction techniques often cause damage to such materials; therefore, alternative non-destructive approaches have been developed [3,22]. The present study demonstrates that materials associated with museum specimens, including filter paper used during fluid filtration as well as the preservation fluid itself, can serve as valuable sources of DNA. Importantly, PCR-amplifiable DNA was successfully recovered from these materials. Moreover, consistent results in ABO genotyping and sex determination were achieved across different sample types—tissue, filter paper, and diluted preservation fluid. The method proposed in this study appears particularly suitable for analyses involving unique and valuable museum collections.

A further important issue to consider when working with museum samples is the degradation and fragmentation of the extracted DNA, especially when the extraction material is filter paper used for conservation fluid filtration or the conservation fluid itself. DNA fragments were expected to be highly degraded, typically ranging from 100 to 150 bp. For this reason, the base pair range was kept short, as in the Jiang et al. [21] method (from 50 to 100 bp to obtain interpretable results). The presented assay for ABO blood group and sex determination, thanks to short amplicon length, was succesfully applied to degraded DNA from 100-year-old museum samples. The assay was successfully applied to filter paper and dialyzed conservation fluid. These findings indicate that the assay may be applied non-destructively on historically important and structurally fragile specimens. Numerous methods for ABO genotyping have been described in the literature. For example, Muro et al. [24] as well as Taki and Kazuhiko [32] investigated PCR-based ABO typing using sequence-specific primers containing intentional mismatches (in 2014), employing a Hot Start Taq DNA polymerase approach. Although their method proved effective for the 27 analyzed samples, it was associated with the occurrence of pseudo-positive signals, which were not observed in the present study. More recent ABO genotyping methods include universal reporter primer systems [33,34] and DNA chip-based approaches [35,36], which offer improved sensitivity for degraded DNA. However, these methods typically require specialized equipment (e.g., microarrays) and are more costly than our multiplex PCR approach, which uses standard laboratory PCR and capillary electrophoresis. Our non-destructive sampling strategy could, in principle, be combined with these more advanced detection systems to further increase throughput.

Maeda et al. [37] applied a PCR-based TaqMan assay to investigate ABO allele frequencies in a Japanese population. Their study identified thirty-one distinct genotypes governed by four common and seven rare alleles; however, the approach was limited due to the high selectivity of the chosen SNP sites.

Bugert et al. [38] utilized PCR with sequence-specific primers for ABO genotyping in paternity testing. Although they demonstrated that ABO genotyping offers advantages over phenotyping in terms of paternity likelihood and exclusion power, it remains insufficient as a standalone method due to the relatively low mutation rate compared with STR markers. Additionally, their study did not address the influence of DNA degradation on PCR-SSP-based ABO genotyping. In the present paper, the PCR-based STR method was used, which was similar to others for both ABO and Amel sex determination [21]. Relatively short amplicons enabled the acquisition of a full profile of the analyzed STR markers, even with difficult material, such as DNA isolated from museum specimens about 100 years old.

Our method should be viewed in the context of recent advances in degraded DNA analysis. MiniSTR panels, such as those developed by Butler et al. (2003) [39], use amplicons of 50–150 bp and are more informative than ABO typing. However, they still require DNA extraction, often from a tissue fragment that has been destroyed. Our non-destructive fluid/filter approach could be easily adapted to such miniSTR primers. Similarly, SNP typing (e.g., Kidd et al., 2014) [40] offers higher multiplexing but demands more complex bioinformatics. Massively parallel sequencing (MPS) of ancient DNA [1,5] provides the richest data but is costly and not universally accessible. Our ABO/Amelogenin multiplex is low-cost, rapid, and non-destructive, making it suitable as a first-line screening tool for museum collections: specimens that yield interpretable ABO profiles are likely to contain sufficient DNA for downstream miniSTR, SNP, or MPS analysis. This two-tier strategy preserves the integrity of rare specimens, especially as new marker systems, such as microhaplotypes, are being developed for highly degraded DNA [39,40].

Moreover, a non-destructive strategy based on the use of associated materials—such as preservation fluid and filter paper used during filtration—proved effective for DNA isolation, yielding PCR-amplifiable DNA of satisfactory quantity and quality. It is important to emphasize that the ABO system alone has lower discriminatory power than STR or SNP panels and is not proposed here as a standalone forensic identification tool. Instead, ABO typing serves three specific purposes in the context of historical museum specimens: (i) it allows for direct comparison with extensive historic serological data, e.g., Mourant’s monographs [24]; (ii) it acts as a proof-of-concept for non-destructive DNA recovery from preservation fluid and filter paper; and (iii) it can be combined with miniSTR or SNP markers in a future multiplex to increase informative value without destroying the specimen. The short amplicon lengths (50–100 bp) used here also make this approach a useful screening test for DNA preservability before committing historically important museum specimens to more expensive and complex analyses such as massively parallel sequencing [1,5].

Limitations of the present study should be acknowledged. First, the ABO system alone provides limited discriminatory power and is not suitable for individual identification. However, as argued above, our primary goal was to establish a non-destructive screening tool. Second, the success rate may vary depending on the original preservation conditions–specimens fixed with formaldehyde or stored under suboptimal conditions may not yield amplifiable DNA even from the preservation fluid. Third, the dialysis step for the conservation fluid is time-consuming (24 h). Future work should explore direct removal of PCR inhibitors using assays or magnetic bead-based purification to shorten processing time. Finally, while we obtained interpretable profiles from 20 museum specimens, larger-scale validation across different preservation media (e.g., formalin-fixed, alcohol-only) would further demonstrate the robustness of the method.

The ABO and Amelogenin multiplex can screen for an individual’s blood group type and gender in a single test using either the 5% Chelex^®^ or NucleoSpin^®^ Tissue Column (MACHEREY-NAGEL (Düren, Germany), Chelex–Bio-Rad Laboratories (Hercules, CA, USA) methods. The method provides a rapid and cost-effective approach for the non-destructive analysis of museum specimens and may be further optimized. The method may provide a potentially applicable methodological approach for all common samples, provided there is sufficient DNA (a minimum of 1.5 ng/mL) in the sample.

It was shown that museum specimens can be an excellent source of DNA and, if formaldehyde was not used for conservation, they remain amenable to PCR amplification. despite considerable DNA degradation. Furthermore, our research to develop an alternative PCR method to the serological method, one that works with degraded DNA samples, combines an already established very short Amelogenin amplicon with PCR sequence-specific primers for ABO genotyping. The multiplex PCR reaction system for ABO and sex determination testing enabled reproducible and cost-effective amplification of all selected loci. Additionally, the assay may provide indirect information regarding the extent of DNAdegradation.

## 5. Conclusions

In this study, we successfully developed and validated a non-destructive PCR-based method for ABO blood group typing and sex determination (Amelogenin) from museum specimens preserved in ethanol-glycerol mixtures for over 100 years. The key conclusions are as follows:

- Non-destructive DNA sources: This study demonstrates that the original preservation fluid and associated filter paper contain amplifiable DNA, allowing complete preservation of the original specimen.
- Cross-validation across three sources: Identical ABO and Amelogenin profiles were obtained from tissue, filter paper, and dialyzed conservation fluid derived from the same specimen, confirming the reliability of the approach.
- Methodological robustness: The use of ultra-short amplicons (50–100 bp for ABO, 208 bp for Amelogenin) enabled reproducible PCR amplification even from highly degraded DNA. Strict contamination controls, following ancient DNA guidelines [2,6,23] and predefined interpretation criteria (Table 2), supported the authenticity and reproducibility of the results.
- Screening tool for museum genomics: Our low-cost, rapid ABO/Amelogenin multiplex can serve as a first-line screening test to assess DNA preservability before committing valuable specimens to costly high-throughput analyses (miniSTR, SNP, or massive parallel sequencing).
- Broader applicability: The same non-destructive strategy can be adapted to other genetic markers (e.g., mitochondrial DNA, microhaplotypes) by simply redesigning primers, provided that amplicon lengths remain short (<150 bp).

Future research should focus on applying this method to larger museum collections and formalin-fixed specimens, and on integrating the non-destructive fluid/filter approach with next-generation sequencing technologies.

## Figures and Tables

**Table 1:** The ABO and Amelogenin amplicons and primers used in this paper: Amelogenin, as designed; ABO-according to Jiang et al. [21].

Locus Amplicons	Dye Label	Primer Alias	Primer Sequence (5′-3′)
0	FAM	Forward	GCGGAAGGATGTCCTCGTGGTAC
		Reverse	CTCGTTGAGGATGTCGATGTT
AB	JOE	Forward	ATGTGGGAAGGATGTCCTCGTGGTGA
		Reverse	CTCGTTGAGGATGTCGATGTT
A	FAM	Forward	ATTGTTCGATTTCTACTACCTGGGGGG
		Reverse	TAGACCATCATGGCCTGGTGG
B	JOE	Forward	AGGACGAGGGCGATTTCTACTACA
		Reverse	TAGACCATCATGGCCTGGTGG
Amel	JOE	Forward	CCTCATCCTGGGCACCCTGG
		Reverse	GCTTGAGGCCAACCATCAGAGC

**Table 2:** Interpretation criteria for ABO and Amelogenin peaks.

Parameter	Criterion
Minimum peak height (RFU)	100 RFU for each allele
Size tolerance	±1 bp from expected size
Required concordance	Some genotype in ≥2 independent PCRs (from same or different source materials)
Positive control	Bloodstain of known ABO group and sex in each run
Negative control	No peaks above 50 RFU in blank and water controls

**Table 3:** The size of ABO and Amelogenin amplicons.

Amplicons	PCR Product Sizes Obtained
0	78/92
AB	82, 92, 97
AA	82, 92
A0	78, 82, 92
BB	82, 97
B0	78, 82, 92, 97
Amel	208/214

## Data Availability

Data is unavailable due to ethical restrictions.

## Abbreviations

The following abbreviations are used in this manuscript:

CODIS	Combined DNA Index System
FAM	6-Carboxyfluorescein
JOE	4,5-dichloro-dimethoxy-fluorescein
